# Femoral Artery Patch Infection and Subsequent Rupture Secondary to Sinus Tract Formation in a Patient With Traumatic Burns

**DOI:** 10.7759/cureus.80462

**Published:** 2025-03-12

**Authors:** Ishan Deshmukh, Constantino G Lambroussis

**Affiliations:** 1 Vascular Surgery, Lake Erie College of Osteopathic Medicine, Elmira, USA; 2 Osteopathic Medicine/Family Medicine, Lake Erie College of Osteopathic Medicine, Elmira, USA

**Keywords:** autologous patch graft, bovine pericardial patch, burn injury, patch infection, peripheral arterial diseases, sinus tracts, vascular repair

## Abstract

Femoral endarterectomies are among the myriad revascularization techniques used to treat peripheral arterial disease. Nonetheless, it is essential to assess individual patient factors to optimize outcomes. While rare, complications such as patch and graft infections or ruptures can occur in these procedures. These complications may be catastrophic, requiring close monitoring and swift intervention to minimize the risk of limb loss or death. This case report describes the successful treatment of a patient who developed an infection in a femoral artery patch, which resulted in rupture due to sinus tract formation following a traumatic burn injury.

## Introduction

Peripheral arterial disease is a prevalent condition affecting up to 12% of the general population, with the femoral artery being a common site of atherosclerotic lesions that can lead to critical limb ischemia [1]. Femoral endarterectomy is a well-established surgical approach that removes obstructing atherosclerotic plaques from the femoral artery, often in conjunction with the placement of a bypass graft or patch repair to restore blood flow [2]. While generally considered a safe and effective procedure, complications such as graft infection and rupture can occur, with an incidence ranging from 1% to 6% [3,4]. These complications can be particularly challenging to manage, as they often require complex surgical interventions and are associated with high rates of morbidity and mortality.

Systemic conditions impairing wound healing and immune function, such as diabetes mellitus and immunosuppressive states, predispose patients to such complications [5,6]. Additionally, local factors such as poor tissue perfusion and the presence of foreign bodies can also increase the risk of infection and subsequent graft failure. Traumatic burns, in particular, can disrupt the normal skin barrier and vascular supply, potentially leading to the development of sinus tracts that may serve as a nidus for infection. Sinus tracts can lead to catastrophic consequences, such as graft rupture, which can be life-threatening and require immediate surgical intervention to prevent limb loss or death [7,8].

Many different arterial reconstruction methods are used in patch repair angioplasty, including direct suture repair of the arterial wall, the use of venous or prosthetic bypass grafts and patches, and the incorporation of biologic material such as bovine pericardial patches [9]. Specific methods of patch angioplasty are associated with varying degrees of risk for complications such as infection and graft/patch failure, and the optimal choice for a given patient requires careful consideration of their unique anatomic features, comorbidities, and the surgeon’s expertise and preference to minimize these risks and maximize long-term durability.

## Case presentation

**Figure 1:**
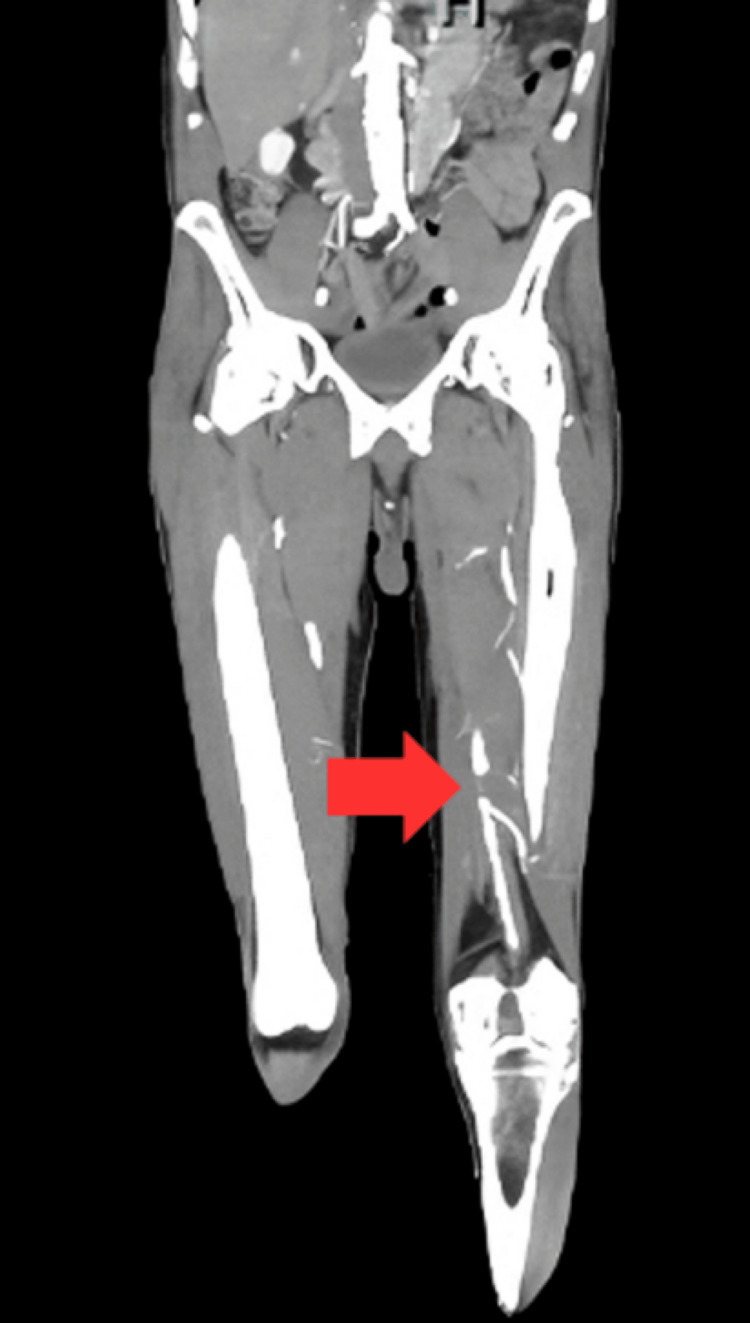
Computed tomography angiography (CTA) of the lower extremity. CTA image of the lower extremity indicating a diffuse atheromatous plaque marked by the red arrow in the superficial femoral artery (SFA). Similar atheromatous lesions were observed in the proximal SFA segment and common femoral artery with a complete occlusion 1.8 cm proximal to the common femoral bifurcation.

A 71-year-old male with a significant smoking history presented to the emergency department with complaints of left leg claudication. The patient had sustained third-degree burns to both lower extremities due to a traumatic injury that occurred eight months prior, which necessitated multiple prior debridement and skin grafting procedures. The burn injuries extended from the level of the malleolus to several centimeters proximal to the knee. At the time of presentation, the burns were in various stages of healing, complicating the assessment of the extent of peripheral arterial disease and collateral circulation based solely on physical examination findings. Notably, low levels of scar tissue from lesser-degree burns were observed at the groin. Ultrasound imaging revealed signs of arterial compromise, which were further corroborated by computed tomography arteriography (CTA) that identified diffuse atherosclerotic lesions. A chronic lesion presenting complete stenosis in the middle segment of the left common femoral artery was noted, along with multiple diffuse lesions in the proximal superficial femoral artery (SFA). A non-emergent vascular surgery consultation was recommended for a potential endarterectomy (Figure 1).

The patient was examined at an outpatient vascular surgery clinic from a different institution and was determined to have an ankle-brachial index (ABI) of 1.2 on the right and 0.79 on the left, but denied pain with ambulation at the time of the consultation. Pulse volume recording studies demonstrated adequate and normal flow in the right lower extremity and reduced flow in the left lower extremity, consistent with significant atherosclerotic disease at the level of the external iliac and common femoral arteries.

The patient was scheduled for a left femoral endarterectomy and patch angioplasty performed by a different surgical team under general anesthesia. The patient was systematically anticoagulated with 5,000 units of heparin, and proximal and distal clamps were placed on the left common femoral arterial segment once isolated. An arteriotomy was made in the anterior arterial wall, and the atheromatous plaque was visualized to have caused a complete occlusion of the left common femoral artery with extension into the origin of the SFA as well as the profunda. The endarterectomy was performed, and once retrograde and anterograde arterial flushing was performed with no debris noted, a bovine pericardial patch was sewn with 5-0 prolene into the arterial wall defect to reconstruct the arterial lumen. The proximal and distal clamps were then removed, and brisk pulsatile flow was reestablished with a monophasic Doppler signal. The patient was monitored with no acute adverse events and reported a significant reduction in claudication for several months post-procedure.

The patient returned to the outpatient vascular surgery clinic one year later with a chief complaint of a persistent, non-healing left malleolar ulceration that had been present intermittently over the past several months, accompanied by some new-onset claudication in his left leg. The patient elected for an elective diagnostic and therapeutic angiography to further evaluate and optimize the arterial flow in the left lower extremity, which demonstrated a patent left common femoral endarterectomy site but noted moderate stenosis in the proximal segment of the left SFA, as well as scattered stenosis of the left popliteal artery and three tandem stenotic lesions in the left anterior tibial artery (AT). Given the non-healing malleolar ulceration and claudication symptoms, the patient underwent a series of endovascular interventions via right femoral arterial access to address the identified stenotic lesions, including angioplasty and stenting with a drug-eluting stent of the left SFA stenosis as well as angioplasty of the left popliteal and tibial arteries under conscious sedation for 120 minutes with a repeat postoperative angiogram showing unencumbered flow through the left SFA, AT, and popliteal arteries confirmed by biphasic signals via doppler ultrasound.

The patient returned to the clinic two months later with a well-healing ulceration on his left lateral malleolus and reduced symptomatic claudication but new complaints of a painful, indurated, red mass in the proximal aspect of his left thigh at the site of the previous endarterectomy and patch angioplasty. A distinct subcutaneous abscess in the left inguinal crease was identified with erythema and induration on physical examination. As reported by the patient, the abscess spontaneously drained mildly sanguineous fluid from its apex several days before presentation and was treated with antibiotics and observation.

In the following weeks, the patient presented to the emergency room multiple times with the complaint of intermittent, large-volume bleeding from the pustule. Although not seen on physical examination during these visits, the patient reported periods of severe bleeding that soaked through dressings and clothing. Due to the patient not having an active, observable bleed and being stable at the time of presentation at the emergency department, the decision was made that the patient should receive an urgent outpatient consultation with the vascular surgeon for further specialized management. An appointment was made within the next few days in which the patient once again presented to the emergency room with an unobserved herald bleed and signs of mild tachycardia.

**Figure 2:**
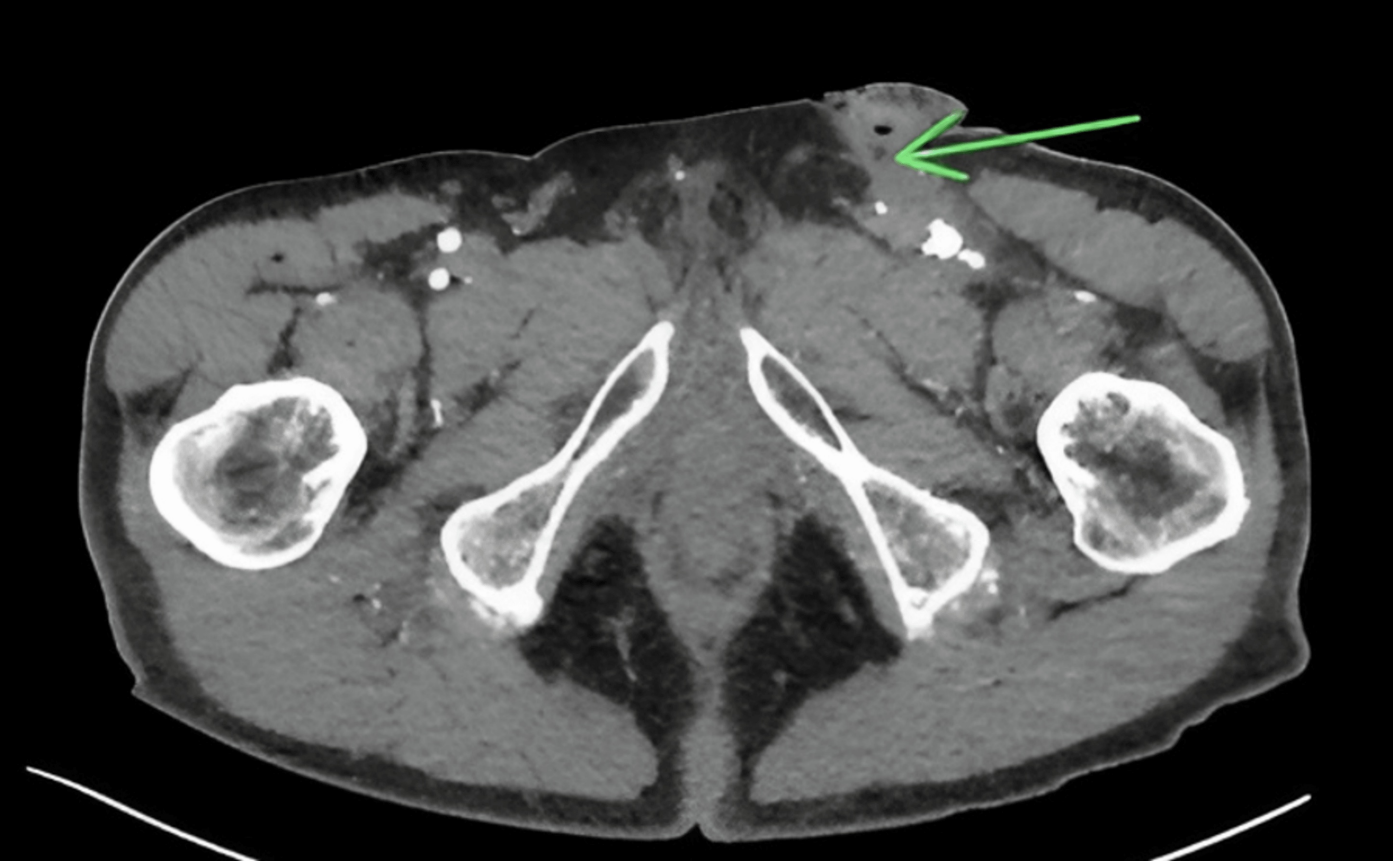
Formation of the sinus tract. A computed tomography angiography transverse image indicating a small sinus tract marked by the green arrow from a post-surgical incision from a prior left femoral artery endarterectomy. The left common femoral artery has significant post-surgical changes consistent with a prior endarterectomy including arterial ectasia measuring 1.3 × 1.3 cm in the axial plane.

The patient was subsequently admitted for further investigation with plans for angiography, MRI, and possible surgical exploration of the sinus tract. The patient received a CTA that indicated the presence of a small sinus tract overlying the incision site from the previous surgical procedure and an MRI was scheduled to further map out the location and dimensions of the sinus tract with surgical exploration scheduled for the following morning (Figure 2).

While the patient was admitted and awaiting surgery, the patient experienced an episode of severe lower extremity bleeding, which became rapidly life-threatening. Emergent exploration revealed that the bleeding was arising from the suppurative sinus tract communicating between the common femoral artery and the overlying skin, requiring immediate surgical intervention. Gross findings of the wound revealed an organized thrombus at the base of the sinus tract, which communicated with the inferior aspect of the bovine pericardium patch, which was noted to have a small defect and a friable appearance.

Ultrasound-guided femoral catheter access was obtained, and an intraoperative arteriogram was performed in which contrast extravasation was noted from the left common femoral arterial patch. Further arteriogram findings indicated high-grade stenosis of the left SFA and profunda femoris. Balloon tamponade using a 6 × 40 mm Mustang balloon (Boston Scientific, Massachusetts, United States) was temporarily achieved at the level of the left external iliac artery, while a superficial incision was created using a 10-blade. Electrocautery was used to dissect down through the subcutaneous tissue and fascial layers, after which a difficult sharp dissection was done using Metzenbaum and tenotomy scissors to expose the underlying vasculature. Proximal control of the left common femoral artery was obtained via vessel loops, after which the balloon tamponade was deflated and removed. An arteriotomy was created, allowing for direct inspection of the inner lumen, which revealed a friable and necrotic arterial wall with a contained leak at the site of the previous bovine patch. The patch was excised out in its entirety with debridement of the sinus tract with culture swabs taken for microbiological studies. The left profunda femoris was directly observed to have complete occlusion during explorative arteriotomy, while the previously placed stent in the SFA was patent and clear of any technical or structural deficits. Fogarty thrombectomy catheters were then passed down the entire length of the left SFA with extensive thrombus extravasation and minimal thrombus burden in the left profunda femoris. Following thrombectomy, systematic heparinization of the left lower extremity arterial tree was performed under proximal and distal control, as well as the application of 6-0 prolene sutures to the distal intimal edge of the prior arteriotomy incision from where the bovine pericardium patch was placed to prevent the formation of an intimal flap. Devitalized intimal tissue directly adjacent to the infected patch in the left common femoral artery was identified and debrided to healthy bleeding tissue to prevent further propagation of the infection and aneurysmal dilatation at the site. To achieve definitive arterial reconstruction, a roughly 10 cm superficial incision was created from the left mid-thigh under ultrasound guidance to mobilize and harvest a length of the ipsilateral greater saphenous vein for the reconstruction of the arterial defect with a new autologous saphenous vein long patch. A 10 cm venous segment was harvested and divided under proximal and distal venous control before insertion into the left common femoral arterial defect with a 5-0 prolene suture and completion of the reconstruction. Arterial flow was re-established and thrombus-free throughout the limb via serial intraoperative angiographic imaging and pulse examination and with a completion arteriogram showing the autologous vein segment to be patent without anastomotic leakage. Post-procedure angiography confirmed the definitive left common femoral artery reconstruction with no evidence of residual stenosis or aneurysmal dilatation. Angiographic evidence of patency of the left SFA and left AT was noted without flow limitation; however, occlusion of the tibio-peroneal trunk was identified with proximal occlusion at the posterior tibial artery and the peroneal artery. Doppler ultrasound and pulse palpation in the operating room confirmed adequate arterial flow distal to the reconstructed segment and the lower extremity, and no evidence of compartment syndrome was observed. Closure of the femoral and saphenectomy site was performed with a combination of absorbable and non-absorbable sutures and staples. All catheters were removed and an Angioseal closure device (Terumo, Tokyo, Japan) was used to close the right femoral arteriotomy site used for angiography purposes.

Microbial analysis of the sinus tract and bovine pericardium patch later confirmed *Staphylococcus aureus* as the definitive infective organism. The patient was transferred to the intensive care unit in a stable condition and immediately started on a targeted course of intravenous antibiotics to address the identified pathogen. In the postoperative period, the patient experienced continued oozing and serosanguineous drainage from the surgical site, as well as a low-grade fever, suggesting persistent infection, which abated with a prolonged three-week course of antibiotics and observation. The patient was discharged and followed up at the outpatient clinic with routine ABIs and reported an improvement in claudication and symptoms at seven months after discharge.

## Discussion

In the management of femoral artery patch infections, timely surgical intervention and aggressive debridement of infected tissue are critical to prevent life-threatening complications and limb loss [10]. Prompt diagnosis and treatment are paramount, as infection of arterial grafts and patches can lead to the rapid development of aneurysmal degeneration, rupture, and exsanguinating hemorrhage. The described case involved a patient presenting with an infected bovine pericardial patch in the left common femoral artery, complicated by a sinus tract and containing an arterial leak. The patient underwent successful surgical removal of the infected patch tissue, debridement, and reconstruction with an autologous greater saphenous vein patch. The decision not to utilize a muscle flap to aid in arterial repair was made due to the concern for introducing an additional site of potential contamination as well as the increased length of time the patient underwent before requiring emergent surgical revascularization that would likely not have tolerated lengthy muscle flap coverage and lead to muscle flap necrosis and patch blowout. The surgical team felt that the increased surgical time and complexity from harvesting and inset of a muscle flap would heighten the likelihood of a subsequent compartment syndrome. Additionally, the patient’s deteriorating clinical condition and prior history of burn trauma suggested they may not have tolerated such an extensive procedure well.

The specific factors that contributed to the sinus tract formation leading to patch infection and subsequent erosion are likely the traumatic third-degree burns and complex limb history with extensive skin grafting the patient had previously, which likely impaired local tissue perfusion and immunity, predisposing to infection.

Skin trauma can disrupt the skin barrier, leading to increased susceptibility to bacterial infection and failed wound healing [11]. Burns destroy the natural skin barrier, leaving a vulnerable surface susceptible to bacterial colonization and chronic inflammation. This, coupled with impaired vascularity from damaged blood vessels, hinders efficient immune response and tissue repair. Similarly, grafted skin, while providing coverage, lacks the robust vascular and lymphatic networks of native skin, further compromising immune surveillance and proper healing.

In particular, skin grafting is associated with an increased risk of infection, given the disruption to the natural skin barrier and lack of intact dermal vascularity and immunity [12]. Burns destroy the natural skin barrier, leaving a vulnerable surface susceptible to bacterial colonization and chronic inflammation. This, coupled with impaired vascularity from damaged blood vessels, hinders efficient immune response and tissue repair. Similarly, grafted skin, while providing coverage, lacks the robust vascular and lymphatic networks of native skin, further compromising immune surveillance and proper healing. In particular, skin grafting is associated with an increased risk of infection, given the disruption to the natural skin barrier and lack of intact dermal vascularity and immunity [13]. The combination of a compromised barrier, impaired circulation, and weakened immunity creates a breeding ground for infection. If this infection becomes localized and walled off, it can form a sinus tract, a channel that burrows into deeper tissues, providing a pathway for persistent infection and potentially leading to complications such as fistulas. The patient’s history of extensive skin grafting likely contributed to the development of the sinus tract and infected bovine pericardial patch in the common femoral artery.

Biomaterial-associated infections, such as in synthetic vascular grafts, can also predispose to these types of complications. For the management of these cases, the key steps typically involve prompt surgical exploration, thorough debridement of all infected and necrotic tissue, removal of the infected material, and definitive arterial reconstruction, often utilizing the patient’s own venous conduit.

Topical antibiotics, appropriate dressing changes, and meticulous wound care are essential, but often not sufficient to prevent or eradicate established infections in the setting of arterial reconstructions. In cases where local infection cannot be controlled by conservative means, surgical debridement and graft/patch replacement remain the gold-standard treatment to prevent catastrophic complications such as hemorrhage, ischemic limb loss, and even death.

## Conclusions

This case report demonstrates the importance of risk stratification in choosing the appropriate arterial conduit material for femoral revascularization, as well as the critical role of early, aggressive surgical management in cases of infected arterial patches. In particular, determining the method of arterial reconstruction that minimizes the risk of future infection is crucial. Surgical teams must remain vigilant for potential complications, particularly in high-risk patients with a history of extensive soft tissue injury, skin grafting, or other factors predisposing to impaired wound healing and infection. Prompt recognition and definitive treatment of infected arterial patches are essential to prevent devastating ischemic complications and limb loss.
